# Editorial: Environmental regulation of feeding, growth, and reproduction in fish: influence of nutrition and physical parameters on the endocrine system

**DOI:** 10.3389/fendo.2023.1281959

**Published:** 2023-09-14

**Authors:** Juan Ignacio Bertucci

**Affiliations:** Centro Oceanográfico de Vigo, Instituto Español de Oceanografía - Consejo Superior de Investigaciones Científicas (IEO-CSIC), Vigo, Spain

**Keywords:** fish endocrinology, growth hormone, HPI axis, fish nutrition, climate change

## Introduction

Within the submerged realm, where aquatic life orchestrates a delicate ballet with environmental cues, the journal *Frontiers in Endocrinology* presents a compelling Research Topic that delves into the intricate symphony of fish biology and its interaction with the environment. This Research Topic embarks on an expedition to elucidate the multifaceted interplay between external stimuli, nutritional dynamics, physiological responses, and the orchestration of endocrine processes within aquatic organisms. In summation, this group of articles attempts to enrich our understanding of the multifaceted interactions between hormones, genetics, nutrition, and the environment in the realm of fish physiology and reproductive biology.

## Revelations in the endocrine landscape

At the heart of this research endeavour lies the profound connection between the endocrine system and fundamental physiological processes, namely feeding, growth, and reproduction, which underpin the life cycle of organisms (1, 2). A central protagonist in this intricate interplay is the growth hormone (GH) and the insulin-like growth factors I and II (IGF-I and IGF-II), pivotal regulators of somatic growth that exert their influence upon diverse target tissues, including muscle and skeletal elements (3, 4). Additionally, the hypothalamic-pituitary-interrenal (HPI) axis emerges as a key architect, orchestrating the modulation of reproduction in response to both intrinsic and extrinsic signals of propitious conditions (5, 6). A third dimension to this narrative unfurls as peptidyl hormones, emanating from cerebral and peripheral realms, choreograph feeding behaviours that resonate harmoniously with metabolic demands (1, 7).

## Deciphering environmental dialogue: the interplay of external signals and endocrine response

The narrative unfolds against a backdrop of external cues that assume the role of conductors, orchestrating change within this intricate biological orchestra. Notably, dietary availability and composition (8–10), and the dynamic physicochemical attributes of water -including temperature, salinity, and pH- (11–14) converge to script the score that reverberates through fish endocrine systems. This intricate ballet enacts the harmonization of internal hormonal pathways with external influences, a dynamic interplay essential for the preservation of fish homeostasis.

## In a changing world: global relevance and prospects

As the world’s population burgeons and fisheries and aquaculture assume pivotal roles in humanity’s sustenance, the exploration of fish endocrine systems becomes profoundly relevant (15, 16). In the face of accelerating environmental modifications wrought by human activities, comprehending the nuanced reverberations of external cues through fish endocrine systems assumes unprecedented significance. The insights gained from this exploration hold the promise of unlocking the key to optimizing aquaculture practices, refining dietary formulations, and enhancing husbandry protocols to bolster yield. Furthermore, the Research Topic’s pursuit of understanding how ongoing global transformations will impact fish populations offers a unique lens through which to glimpse the unfolding narrative of aquatic life.

## Exploring the collected overture: highlights of contributing articles

Embedded within this research compendium are the articles that together craft an intricate overture. “*Neuroendocrine Regulation of Plasma Cortisol Levels During Smoltification and Seawater Acclimation of Atlantic Salmon*” navigates the cortisol dynamics of life stage transitions (Culbert et al.). This transition involves changes in their ability to manage salt levels in their bodies. The study focuses on understanding the control of cortisol through the brain’s HPI axis. “*Analysis of circRNA and miRNA expression profiles in IGF3-induced ovarian maturation in spotted scat (Scatophagus argus)*” peers into the realm of ovarian maturation. While IGF3’s role in teleost ovarian maturation is known, its molecular regulation is not well understood (Li et al.). Circular RNAs (circRNAs) and microRNAs (miRNAs), important in various biological processes including reproduction, are studied in relation to IGF3-induced ovarian maturation. “*Food deprivation differentially modulates gene expression of LPXRFa and kisspeptin systems in the brain-pituitary axis of half-smooth tongue sole (Cynoglossus semilaevis)*” unravels the genetic tapestry of food deprivation’s effects (Wang et al.). Authors investigate how nutritional status affects the expression of two key neuroendocrine systems, LPXRFa (also known as gonadotropin-inhibitory hormone or GnIH) and kisspeptin (Kiss), which play vital roles in regulating the reproductive axis in many vertebrates, including fish. “*Knock-out of vasotocin reduces reproductive success in female zebrafish, Danio rerio*” sheds light on the significance of vasotocin in female teleost reproduction (Ramachandran et al.). Finally, the review article entitled “*The effect of environmental stressors on growth in fish and its endocrine control*” delves into the complex interplay among somatic growth, the endocrine system, feeding regulation, and environmental stressors. It underscores the significance of grasping these connections to fully appreciate how environmental shifts affect fish populations and ecosystems (Canosa et al.).

## Final considerations

Through the pages of *Frontiers in Endocrinology*, this Research Topic unfurls as an intellectual voyage. Each article encapsulates a profound exploration of the dance between fish biology and the surrounding milieu. This Research Topic of insights collectively illuminates the intricate symphony of endocrine responses harmonizing with external signals, creating an enduring melody that resonates within the aquatic realm. As we navigate the uncharted waters of aquatic life, these insights emerge as guiding beacons, illuminating our way through the captivating tapestry of fish physiology and its profound liaison with the aquatic stage.

## Author contributions

JB: Writing – original draft, Writing – review & editing.

## References

[1] Bertucci JI Blanco AM Sundarrajan L Rajeswari JJ Velasco C Unniappan S. Nutrient regulation of endocrine factors influencing feeding and growth in fish. Front Endocrinol (Lausanne) (2019) 10:83. doi: 10.3389/fendo.2019.00083 30873115 PMC6403160

[2] Canosa LF Bertucci JI. Nutrient regulation of somatic growth in teleost fish. The interaction between somatic growth, feeding and metabolism. Mol Cell Endocrinol (2020) 518:111029. doi: 10.1016/j.mce.2020.111029 32941926

[3] Pérez-Sánchez J Simó-Mirabet P Naya-Català F Martos-Sitcha JA Perera E Bermejo-Nogales A. Somatotropic axis regulation unravels the differential effects of nutritional and environmental factors in growth performance of marine farmed fishes. Front Endocrinol (Lausanne) (2018) 9:687. doi: 10.3389/fendo.2018.00687 30538673 PMC6277588

[4] Dai XY Zhang W Zhuo ZJ He JY Yin Z. Neuroendocrine regulation of somatic growth in fishes. Sci China Life Sci (2015) 58:137–47. doi: 10.1007/s11427-015-4805-8 25655896

[5] Faught E Aluru N Vijayan MM. The molecular stress response. Fish Physiol (2016) 35:113–66. doi: 10.1016/B978-0-12-802728-8.00004-7

[6] Gorissen M Flik G. The endocrinology of the stress response in fish: an adaptation-physiological view. Fish Physiology. Academic Press (2016) 75–111. doi: 10.1016/B978-0-12-802728-8.00003-5

[7] Volkoff H. The neuroendocrine regulation of food intake in fish: A review of current knowledge. Front Neurosci (2016) 10:540. doi: 10.3389/fnins.2016.00540 27965528 PMC5126056

[8] Bertucci JI Blanco AM Canosa LF Unniappan S. Glucose, amino acids and fatty acids directly regulate ghrelin and NUCB2/nesfatin-1 in the intestine and hepatopancreas of goldfish (Carassius auratus). vitro. Comp Biochem Physiol Part A Mol Integr Physiol (2017) 206:24–35. doi: 10.1016/j.cbpa.2017.01.006 28089858

[9] Zhang J Zhao N Sharawy Z Li Y Ma J Lou Y. Effects of dietary lipid and protein levels on growth and physiological metabolism of Pelteobagrus fulvidraco larvae under recirculating aquaculture system (RAS). Aquaculture (2018) 495:458–64. doi: 10.1016/J.AQUACULTURE.2018.06.004

[10] Hemre GI Mommsen TP Krogdahl ÅChecktae. Carbohydrates in fish nutrition: Effects on growth, glucose metabolism and hepatic enzymes. Aquac Nutr (2002) 8:175–94. doi: 10.1046/j.1365-2095.2002.00200.x

[11] Reinecke M. Influences of the environment on the endocrine and paracrine fish growth hormone-insulin-like growth factor-I system. J Fish Biol (2010) 76:1233–54. doi: 10.1111/j.1095-8649.2010.02605.x 20537012

[12] Boeuf G Payan P. How should salinity influence fish growth? Comp Biochem Physiol C Toxicol Pharmacol (2001) 130:411–23. doi: 10.1016/S1532-0456(01)00268-X 11738629

[13] Teles AO Couto A Enes P Peres H. Dietary protein requirements of fish – a meta-analysis. Rev Aquac (2020) 12:1445–77. doi: 10.1111/raq.12391

[14] Poloczanska ES Burrows MT Brown CJ García Molinos J Halpern BS Hoegh-Guldberg O. Responses of marine organisms to climate change across oceans. Front Mar Sci (2016) 3:62. doi: 10.3389/fmars.2016.00062

[15] Boyd CE D’Abramo LR Glencross BD Huyben DC Juarez LM Lockwood GS. Achieving sustainable aquaculture: Historical and current perspectives and future needs and challenges. J World Aquac Soc (2020) 51:578–633. doi: 10.1111/JWAS.12714

[16] Buck BH Troell MF Krause G Angel DL Grote B Chopin T. State of the art and challenges for offshore Integrated multi-trophic aquaculture (IMTA). Front Mar Sci (2018) 5:165. doi: 10.3389/FMARS.2018.00165

